# The Methyltransferase AflSet1 Is Involved in Fungal Morphogenesis, AFB1 Biosynthesis, and Virulence of *Aspergillus flavus*

**DOI:** 10.3389/fmicb.2020.00234

**Published:** 2020-02-18

**Authors:** Yaju Liu, Mengjuan Zhang, Rui Xie, Feng Zhang, Sen Wang, Xiaohua Pan, Shihua Wang, Zhenhong Zhuang

**Affiliations:** Key Laboratory of Pathogenic Fungi and Mycotoxins of Fujian Province, Key Laboratory of Biopesticide and Chemical Biology of Education Ministry, and School of Life Sciences, Fujian Agriculture and Forestry University, Fuzhou, China

**Keywords:** *Aspergillus flavus*, histone methyltransferase, AflSet1, morphology, virulence

## Abstract

The filament fungal pathogen, *Aspergillus flavus*, spreads worldwide and contaminates several important crops. Histone posttranslational modifications are deeply involved in fungal development and virulence, but the biological function of the histone methyltransferase AflSet1 in *A. flavus* is still unknown. In the study, *Aflset1* deletion strain was constructed through homologous recombination, and it was found that AflSet1 up-regulates hyphae growth, and promotes conidiation by sporulation regulation genes: *abaA* and *brlA*. It was also found that AflSet1 involves in sclerotia formation and AFB1 biosynthesis via sclerotia related transcriptional factors and orthodox AFB1 synthesis pathway, respectively. Crop models revealed that AflSet1 plays critical roles in colonization and AFB1 production on crop kernels. Lipase activity analysis suggested that AflSet1 affects fungal virulence to crops via digestive enzymes. Stresses tests revealed that AflSet1 is deeply involved in fungal resistance against osmotic, oxidative and cell membrane stress. The preparation of N_SET, SET domain deletion mutants and H988K mutant revealed that both domains play critical roles in fungal development and AFB1 production, and that H988 is very important in executing biological functions on morphogenesis and AFB1 synthesis. Subcellular location analysis revealed that AflSet1 is stably accumulated in nuclei in both spore germination and hyphae growth stages, even under the stress of SDS. Through immunoblot analysis, it was found that AflSet1 methylates H3K4me2 and me3 as well as H3K9me2. This study provides a solid evidence to discover the biological functions of histone methyltransferase in pathogenic fungi.

## Introduction

As a soil saprophyte, *Aspergillus flavus* spreads worldwide and contaminates several agriculture important crops, such as rice, corn, peanut and cotton, before and after harvest. The fungus endangers the health of animal through its toxic secondary metabolites (especially aflatoxins) by causing aflatoxicosis or hepatocellular carcinoma. By invading growth in animal tissues, the pathogenic fungus also causes aspergillosis, which is always lethal to immunocompromised patients (Amaike and Keller, 2011). The fungus produces a serial of mycotoxins, such as aflatoxins, cyclopiazonic acid and aflatrem. Aflatoxins are a class of polyketide derived carcinogens, which were firstly found after an outbreak of Turkey X disease happened in England in early 1960s (Amaike and Keller, 2011). In case low level of aflatoxins is daily ingested, this can cause chronic aflatoxicosis which may stimulate the development of liver cancer, and high level ingestion of aflatoxins can cause acute aflatoxicosis, even death like what happened in Turkey X disease. Among aflatoxins, aflatoxin B1 (AFB1) is known to be the most toxic natural chemical compound produced by *A. flavus*, and it is reported that the toxicity of AFB1 is 10 times than that of cyanide (Seenappa and Kempton, 1980). Therefore, it is important to attenuate the detriment of *A. flavus* and its secondary metabolites, especially AFB1, to crops and human beings.

The morphogenesis, mycotoxin biosynthesis and virulence of filament fungus were found to be regulated by a series of orthodox regulators. AflR is necessary for aflatoxin biosynthesis by up-regulating the activity of most genes in the aflatoxin pathway (Chang et al., 1995; Ehrlich et al., 1999). The global regulator VeA is involved in light response, sexual propagation and secondary metabolism in *A. nidulans* (Bayram et al., 2010). The VelB-VeA-LaeA (velvet) complex is critical for conidiation and aflatoxin biosynthesis in *A. flavus* (Chang et al., 2013). The master transcription factor MtfA involves in the secondary metabolism, conidiation and sclerotia formation in *A. flavus* (Zhuang et al., 2016). So regulation mechanisms of *A. flavus* development and virulence are complicated and urgently need to be further elucidated. Few is known about the role of epigenetics in the pathogenicity of *A. flavus*. Epigenetic modification regulates the expression levels of genes by the switch between euchromatic and heterochromatic state of DNA regions, in which the nucleotide sequence is stable, but the epigenetic signatures are changeable (Angarica and Sol, 2017). As an important marker of epigenetics, the posttranslational modification of histones deeply participates in many genetic regulations, such as transcriptional regulation, DNA replication and repair, and chromosome segregation (Freitag, 2017). Set domain proteins were firstly discovered from *Drosophila melanogaster*, including three proteins: Su (var) 3–9, E(z), and Trx (Freitag, 2017). Related studies in mammals, insects, fungi, and plants have preliminarily shown that Set domain proteins are a class of histone lysine methyltransferases (Rea et al., 2000; Tamaru and Selker, 2001; Jackson et al., 2002; Allis et al., 2007; Zhao et al., 2019). As a set domain containing methyltransferase, Set1 is closely binding with 60 (Bre2), Cps50 (Swd1), Cps30 (Swd3) and Cps40 in Y-shaped COMPASS (complex of proteins associated with Set1) (Qu et al., 2018). The homolog of Bre2 in *Saccharomyces cerevisiae*, CclA was found to play a critical role in H3K4 trimethylation catalyzed by COMPASS complex in *Colletotrichum higginsianum* (Dallery et al., 2019). The absence of CclA significantly reduces hyphae growth, conidiation and conidium germination, and attenuates the virulence of the fungus on its plant host, but greatly enriches the profile of its secondary metabolites (Dallery et al., 2019). FgSet1 is involved in the methylation of H3K4 in *Fusarium graminearum*, and plays an important role in mycelium growth, mycotoxin biosynthesis, and virulence in the fungus (Liu et al., 2015).

Since histone methylations play critical roles in the morphogenesis, secondary metabolism and virulence of pathogenic fungi, it is of importance to illustrate the biological function of histone methyltransferase in plant and animal pathogen, *A. flavus*. We located an FgSet1 homologous protein – AflSet1 (62.11% Ident, 83% Query Cover) in the pathogenic fungus *A. flavus* from NCBI^1^ through bioinformatics analysis, and our study is designed to clarify the biological function of AflSet1 (Gene bank No. in NCBI: AFLA_037970) in morphogenesis, mycotoxin biosynthesis and the virulence of *A. flavus*.

## Materials and Methods

### Fungal Strains and Primers

All fungal strains used in the work were listed in Table 1. The primers used in this study were presented in Supplementary Tables S1, S2. The medium PDA (39 g/L potato dextrose agar, from BDDifco, Franklin, NJ, United States), YES (2% yeast extract, 150 g/L sucrose, 1 g/L MgSO_4_∙7H_2_O), WKM (Wickerham medium, 2 g/L yeast extract, 3 g/L peptone, 5 g/L cornteep solids, 2 g/L dextrose, 30 g/L sucrose, 2 g/L NaNO_3_, 1 g/L K_2_HPO_4_∙3H_2_O, 0.5 g/L MgSO_4_∙7H_2_O, 0.2 g/L KCl, 0.1 g/L FeSO_4_∙7H_2_O) and Recovery medium (372.4 g/L sucrose, 3g/L NaNO_3_, 1 g/L K_2_HPO_4_, 0.5 g/L KCl, 0.5 g/L MgSO_4_, 0.01 g/L FeSO_4_, 10 mM Ammonium tartrate, 0.5% Agar) were prepared for fungal culture, and 15 g/L agar was added for solid medium. 1 mg/mL uracil and uridine were added for the fungal strains with the auxotrophic marker (*pyrG*-) (Hu et al., 2018).

**TABLE 1:** The fungal strains used in this study.

Fungal strains	Genotype description	References
CA14	Δ*pyrG*, Δ*ku70*	Purchased from FGSC
Control(Ctrl)	CA14, *pyrG*+	Prepared in our lab
Δ*Aflset1*	CA14, Δ*Aflset1:pyrG*	This study
*Aflset1*^ΔN_SET^	CA14, *Aflset1*^Δ^*^N_SET^:pyrG*	This study
*Aflset1*^ΔSET^	CA14, *Aflset1*^ΔSET^*:pyrG*	This study
*Aflset1*^H988K^	CA14, *Aflset1*^H988K^*:pyrG*	This study
Δ*Aflset1*-Com	Δ*pyrG*,Δ*ku70*, *pyrG*+	This study

*CA14 stands for CA14 Δ*pyrG* Δ*ku70* in the paper.*

### Preparation of Mutant Strains

**FIGURE 1:**
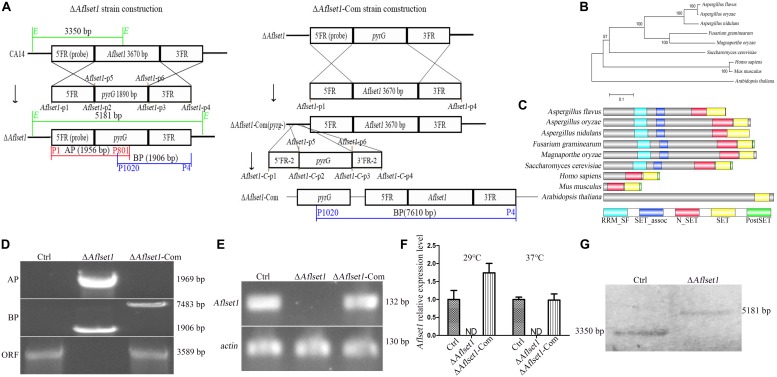
Bioinformatics analysis of AflSet1 and the construction of *Aflset1* deletion and complementary strains. **(A)** The strategy for *Aflset1* deletion and complementary strains. FR is for flanking region. **(B)** Construction of phylogenetic relationship among 9 AflSet1 homologs (from *A. flavus*, *A. oryzae*, *A. nidulans*, *F. gramincarum*, *M. oryzae*, *S. cerevisiae, M. musculus*, *H. sapiens*, and *A. thaliana*) with MEGA5.0. **(C)** The domains of AflSet1 were characterized by software SMART, and the identified domains were visualized using IBS 1.0. **(D)** The constructed Δ*Aflset1* andΔ*Aflset1*-Com strains were validated by PCR with genomic DNA as template. DNA fragment AP was amplified with primer p1 and p801, BP with primer p1020 and p4, and ORF with primer p9 and p10 as shown in the strategy panel **(A)**. **(E)**The *Aflset1* ORF in the above fungal strains was further tested by qPCR with primer *Aflset1*-q-F and *Aflset1*-q-R using cDNA as template, and *actin* was utilized as inner reference. **(F)** The expression level of *Aflset1* in above fungal strains at 29°C and 37°C was monitored with qRT-PCR. **(G)** *Aflset1* mutant (Δ*Aflset1*) was identified by Southern-blotting analysis. Genomic DNA from Ctrl and Δ*Aflset1* strains were digested by *EcoR1* (*E*) and hybridized with a 1.35 kb probe (the 5’- flanking region of *Aflset1*), and the probe was amplified with primers *probe*-F and *probe*-R.

The *Aflset1* gene deletion strains were prepared according to the method of homologous recombination (Yang et al., 2019), and named as Δ*Aflset1*. 1168 bp 5′- flanking region (with primer *Aflset1*-p1 and *Aflset1*-p2) and 889 bp 3′- flanking region (primer *Aflset1*-p3 and *Aflset1*-p4) of *Aflset*1 were amplified, and fused together with *pyrG* by using nesting primer *Aflset1*-p7 and *Aflset1*-p8 according to the strategy scheme shown in Figure 1A. The fusion production was used to transform *A. flavus* protoplasts (CA14) to generate *Aflset1* deletion mutant (Δ*Aflset1*). Δ*Aflset1* strain was tested and confirmed with diagnostic PCR, q-PCR and southern-blotting analysis. These mutant strains were confirmed by DNA sequencing in BioSune Biotechnology (Shanghai, China) Co., Ltd. The *Aflset1* complementary strain (Δ*Aflset1*-Com) was constructed by the protocol provided by Hu et al. (2018) with minor modification. The *pyrG* in Δ*Aflset1* was replaced by 5′-flanking region-*Aflset1*-3′-flanking region (amplified with primer *Aflset1*-p7 and *Aflset1*-p8) under the stress of 2 mg/mL 5-FOA (5-fluoroorotic acid). Then, *pyrG* gene was inserted into the transformants at N-terminal of *Aflset1* gene by homologous recombination with the fusion production amplified by *Aflset1*-*C*-p1 and *Aflset1*-*C*-p4 primers as shown in the strategy scheme in Figure 1A. Finally the Δ*Aflset1*-Com strain was further verified by diagnostic PCR and q-PCR. The preparation of *Aflset1*^ΔN_SET^, *Aflset1*^ΔSET^, and *Aflset1*^H988K^ mutants were carried out according to the method of homologous recombination mentioned above. For *Aflset1*^ΔN_SET^ mutant strain preparation, four DNA fragments were amplified with four pairs of primers: *Aflset1*-pN1 and *Aflset1*-pN1-R, *Aflset1*-pN2 and *Aflset1*-pN2-R, *pyrG*-F and *pyrG*-R, and *Aflset1*-pN4-F and *Aflset1*-p4, respectively (Supplementary Table S1), and these four amplified fragments were fused together with nesting primer *Aflset1*-pN8-F and *Aflset1*-p8 (Supplementary Table S1). To generate *Aflset1*^ΔSET^ mutant, three DNA fragments were amplified with three pairs of primers: *Aflset1*-pN8-F and *Aflset1*-pS1-R, *Aflset1*-*pyrG*-F and *pyrG*-R, and *Aflset1*-PS-4-F and *Aflset1*-p4 (Supplementary Table S1), and they were fused together by nesting primers: *Aflset1*-PS-8-F and *Aflset1*-p8 (Supplementary Table S1). In *Aflset1*^H988K^ construction, four DNA fragments were amplified with four pair of primers: *Aflset1*-H988K-1-F and *Aflset1*-H988K-1-R, *Aflset1*-H988K-2-F and *Aflset1*-H988K-2-R, *pyrG*-F and *pyrG*-R, and *Aflset1*-H988K-3-F and *Aflset1*-H988K-3-R (Supplementary Table S1), and these DNA fragments were purified and fused together by primers: *Aflset1*-H988K-1-F and *Aflset1*-H988K-3-R. Then, above domains or point mutant fungal strains were prepared by transforming *A. flavus* CA14 protoplasts with above purified fusion-PCR production, respectively. Finally, the resultant transformants were selected with Recovery medium, and further verified by sequencing in BioSune (Shanghai, China).

### Bioinformatics Analysis

The homologs of Set1 (from *A. flavus*, *A. oryzae*, *A. nidulans*, *Fusarium gramincarum*, *Magnaporthe oryzae*, *S. cerevisiae, Mus musculus*, *Homosapiens*, and *Arabidopsis thaliana*) were downloaded from NCBI (see text footnote^1^), and their evolutionary relationship was analyzed with MEGA5.0. The domains of AflSet1 were located by DOG2.0^2^, and further visualized by IBS 1.0 (Hu et al., 2018).

### Phenotype Analysis

The same amount (10^4^) of spores was inoculated on Petri dishes with 15 mL corresponding medium (PDA and YES agar for conidiation, WKM media for sclerotia formation) under 37°C. The growth rates of fungal strains were reflected by the diameters of the fungal colonies. To count the conidia number, three agar cores (1 cm in diameter) were drilled at equal distance along the radius of each fungal colony, and after all these agar cores were collected into one 5 mL falcon tube containing 3 mL water, and the falcon tube was vortexed, then, the number of spores was calculated with hemocytometer under microscope. The number of sclerotia was counted under anatomical lens after cultured with WKM media.

### Real-Time Quantitative Reverse Transcription PCR (qRT-PCR) Analysis

The analysis of qRT-PCR was performed following the protocol that was formerly described by Nie et al. (2016) with minor modification. Spores were inoculated with corresponding medium (PDA and YES agar for conidiation, WKM media for sclerotia formation) for 48 h, and the fungal hyphae were collected and ground into powder in liquid nitrogen. Then, total RNA was extracted with RNA isolation kit (Promega, United States). The first strand cDNA was synthesized with Revert Aid First-strand cDNA Synthesis Kit (TransGen Biotech, China) from 3 μg total RNA. The primers for the analysis were listed in Supplementary Table S2. Each experiment was repeated at least five times.

### Analysis of AFB1 Production

The analysis of AFB1 was performed according to the method previously described by Hu et al. (2018). AflSet1’s role in AFB1 bio-synthesis was detected by inoculating10^7^/mL conidia in 10 mL liquid YES medium at 29°C for 6 days. The extracted AFB1 was further analyzed with TLC (thin layer chromatography), and observed with GBox XT4 Chemiluminescence and Fluorescence Imaging System (Gene Company Limited, Shanghai, China) at a wavelength of 302 nm. Gene Tools (software version: 4.03.05.0) was used to determine the relative amount of AFB1 in each sample according to the results of TLC through comparison with the standard AFB1.

### Analysis of Fungal Ability to Colonize on Kernels

The ability of *A. flavus* to colonize on kernels was analyzed according to the protocol given by Yuan et al. (2018) with minor modification. After sterilized by 0.05% sodium hypochlorite, the kernels were inoculated with 5 × 10^5^ fungal spores. Then, the kernels were dried and cultured at 29°C for 6 days. Finally, the number of conidia was calculated with hemocytometer, and AFB1 was extracted from these kernels with chloroform for 3 h, and analyzed by TLC.

### Subcellular Localization

To locate Aflset1 inside fungal cells, *mcherry* gene was fused with *Aflset1* gene by homologous recombination. Spores (10^4^) were inoculated in liquid YES for 12 h under 37°C, then the hyphae were collected, and the subcellular location of mcherry-AflSet co-expression proteins (emitting red fluorescence) was identified under laser confocal scanning microscope (LeicaSP8). The nuclei of fungal hyphae were located by DAPI staining.

### Stress Tests

To evaluate the roles of AflSet1 in fungal resistance against environmental stresses, 10^4^ fungal spores were inoculated on PDA based medium at 37°C for 3 to 5 days with corresponding inhibitor, including oxidative stress inhibitor H_2_O_2_, plasma membrane inhibitor sodium dodecyl sulfate (SDS), osmotic stress factors NaCl and KCl, cell wall inhibitor calcofluor white stain (CFW) and DNA stress factor methylmercuric sulfate (MMS). The diameters of fungal colonies were measured 3 days (for NaCl and KCl stresses) or 5 days (for H_2_O_2_, SDS, CFW, and MMS stresses) after inoculation. Finally, the inhibition rates were calculated by the following formula: inhibition rate = (colony diameter without inhibitor – colony diameter with inhibitor)/colony diameter without inhibitor.

### Western-Blotting Analysis

Western-blotting Analysis was performed to identify the histone methylation sites catalyzed by AflSet1. After fungal spores (5 × 10^5^) were inoculated in liquid YES at 37°C for 48 h, the hyphae were collected and grounded into powder in liquid nitrogen, and the protein samples were immediately extracted by RIPA Lysis Buffer (TransGen Biotech, Beijing). The protein samples were analyzed with SDS-PAGE, after that the PVDF membrane was activated with methanol for 30 s. Then, proteins in the gel were transferred onto PVDF membrane at a current of 300 mA for 50 m, and the membrane was further blocked with 5% skim milk powder in TBST solution (8 g/L NaCl, 2.42 g/L Tris, 1 mL Tween-20, pH7.6) for 1 h. Then the membrane was soaked in 1:1000 diluted 1st antibody (the murine monoclonal antibodies against H3K4me1 to me3, H3K9me1 to me3 and H3K36me1 to me3, respectively), in TBST solution with 5% skim milk at 4°C overnight. Following that, the membrane was eluted three times with 20 mL of TBST for 7 m each time, then the membrane was soaked into 1:5000 diluted goat anti-mouse antibody dilution at room temperature for 1 h. Finally, the membrane was washed with TBST for three times, and the color was developed with equal volume of HRP Substrate Luminol Reagent (Immobilon^TM^ Western) and HRP Substrate Peroxide Solution (Immobilon^TM^ Western), and photographed in GBox XT4 Chemiluminescence and Fluorescence Imaging System (Gene Company Limited, Shanghai, China) (Zhuang et al., 2011).

### Statistical Analysis

In the work, all data were presented by means ± SD (standard deviation). One-way ANOVA was used in the study to determine the statistical differences, and it was accepted as statistical significant when a *p*-value is less than 0.05. All experiments in the study were repeated at least three times.

## Results

### Bioinformatics Analysis and Mutant Strains Construction

The evolutionary relationship of 9 Set1 homologous proteins (from *A. flavus*, *A. oryzae*, *A. nidulans*, *F. gramincarum*, *M. oryzae*, *S. cerevisiae*, *M. musculus*, *H. sapiens*, and *A. thaliana*) was analyzed with MEGA5.0. It showed that AflSet1 from fungi was classified into one group, in which the highest similarity (98.51% Identity, 99% Query Cover) was identified between *A. flavus* and *A. oryzae*, and the lowest similarity (77% Identity, 29.06% Query Cover) was found between *A. flavus* and *S. cerevisiae* (Figure 1B). The domains in AflSet1 were further identified using SMART, and visualized by IBS 1.0. A RRM_SF, a SET_assoc, a N_SET, a SET and a PostSET domain were found from these homologs, and all these domains could be found in the homologs from fungi (Figure 1C). The *Aflset1* gene deletion mutant (Δ*Aflset1*) was constructed by the method of homologous recombination with the production of overlapping PCR (5′- flanking region -*pyrG*-3′-flanking region), and the candidate *Aflset1* deletion transformants were tested by PCR to amplify AP, BP fragments and the ORF of *Aflset1*, as shown in Figures 1A,D and Supplementary Figure S1. By diagnostic PCR, it was found that a 3.6 kb fragment of *Aflset1* ORF was only amplified from wild type strain (Ctrl) and complementary strain (Δ*Aflset1-*Com), and both 1.95 kb AP and 1.9 kb BP fragments could only be amplified from *Aflset1* mutant, while a 7.6 kb fragment was amplified from Δ*Aflset1-*Com (Figure 1D and Supplementary Figure S1). According to the strategy scheme (Figure 1A), these results suggested that the Δ*Aflset1* and Δ*Aflset1-*Com have been successfully constructed in the study. After cDNA was prepared from total RNA, an amplified 141 bp DNA fragment inside *Aflset1* ORF was detected in Ctrl and Δ*Aflset1-*Com strains, but not in Δ*Aflset1* by qPCR analysis as shown in Figure 1E (actin was chosen as inner reference). The qRT-PCR analysis on the expression level of *Aflset1* in these fungal strains was performed, and the results confirmed that the activity of *Aflset1* could not be detected in Δ*Aflset1*, but it was recovered in Δ*Aflset1-*Com strain compared with Ctrl strain (Figure 1F). According to the strategy scheme in Figure 1A, the result of southern-blotting analysis further verified that Δ*Aflset1* strain has been constructed (Figure 1G). The above results indicated that Δ*Aflset1* and Δ*Aflset1-*Com strains have been successfully generated in the study.

### AflSet1 Is Involved in the Growth and Conidiation of *A. flavus*

**FIGURE 2:**
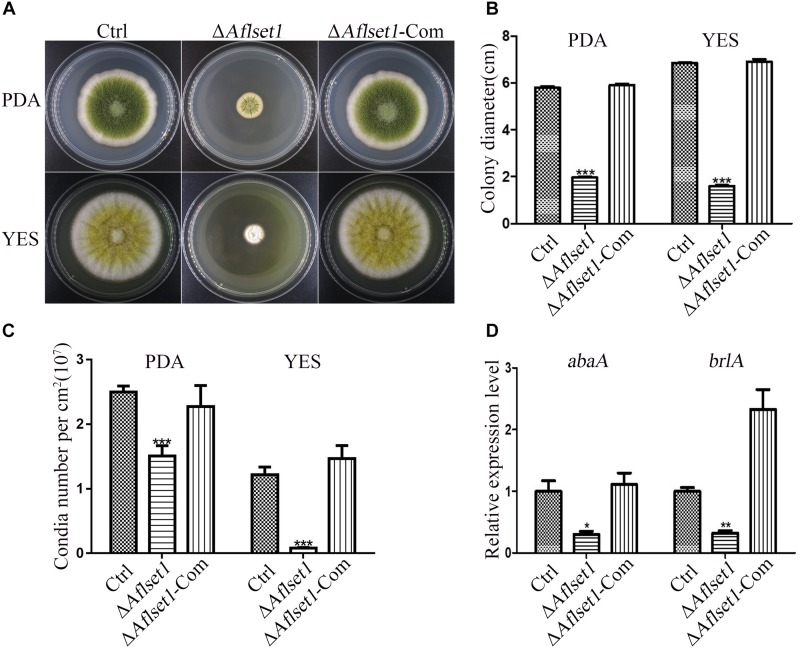
AflSet1 is involved in hyphae growth and conidiation in *A. flavus*. **(A)** The fungal strains were point-inoculated on PDA media for 6 days and YES agar for 4 days at 37°C. **(B)** The colony diameters were measured and represented with column graph according to the result of **(A)** panel. **(C)** The column graph showed the conidia number of each fungal strain on PDA and YES medium. **(D)** The expression level of transcriptional factor *abaA* and *brlA* genes in conidiation. The “*,” “**,” and “***” represents significant difference levels: *p* < 0.01, *p* < 0.005 and *p* < 0.001, respectively. All experiments were implemented with three biological replicates, and repeated at least three times.

The spores of all the *A. flavus* strains (Ctrl, Δ*Aflset1* and Δ*Aflset1*-Com) were point inoculated on PDA and spores were left to grow for 6 days at 37°C, and the same procedure was repeated on YES agar and spores were left to grow for 4 days at 37°C as depicted in Figure 2A. It was found that the absence of AflSet1 from *A. flavus* dramatically decreased the growth rate of hyphae (only about 2 cm in colony diameter to both media) compared to both Ctrl and Δ*Aflset1*-Com (reach about 6 cm in PDA medium, and about 7 cm in YES medium) as shown in Figures 2A,B (*p* < 0.001). The conidia number was further calculated with hemocytometer, and the results showed the deletion of *Aflset1* gene significantly reduced the sporulation ability of the fungus. As shown in Figures 2A,C, Δ*Aflset1* strain only produced about 0.1 to 1.5 × 10^7^ conidia/cm^2^ on PDA medium and YES medium, but the conidia number is up to about 1.5 to 2.5 × 10^7^/cm^2^ in Ctrl and Δ*Aflset1*-Com strains on both media. To explore the pathway by which AflSet1 regulates sporulation, spores (5 × 10^5^) of above fungal strains are inoculated in PDA medium at 37°C for 48 h, and the fungal hyphae were collected and grounded into powder in liquid nitrogen, followed by the total RNA extraction and cDNA synthesis by reverse transcription. The following qRT-PCR analysis showed that expression levels of both *abaA* and *brlA* genes, the key transcriptional factors in sporulation regulation, were remarkably decreased when *Aflset1* was deleted compared to Ctrl and Δ*Aflset1*-Com strains (Figure 2D). All these results indicated that AflSet1 positively regulates the growth of colony, and up-regulates conidiation through *abaA* and *brlA* mediated pathway.

### AflSet1 Is Critical in Sclerotia Formation in *A. flavus*

**FIGURE 3:**
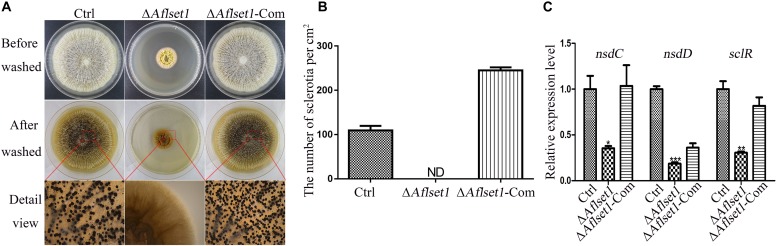
AflSet1 is necessary in sclerotia formation in *A. flavus*. **(A)** All three fungal strains (Ctrl, Δ*Aflset1* and Δ*Aflset1*-Com) were point inoculated on Wickerham medium and grown for 1 week at 37°C. **(B)** The column graph showed the sclerotia number from the results of **(A)** panel. **(C)** The expression levels of the key positive regulator genes (*nsdC*, *nsdD*, and *sclR*) in the formation of sclerotia in *A. flavus*. The experiment was carried out with three biological replicates, and repeated three times.

To assess the role of AflSet1 in sclerotia formation, the fungal strains were inoculated on Wickerham medium for 1 week, and the number of sclerotia was calculated under anatomical lens after hyphae and spores were washed away with 70% ethanol (Figure 3A). The result showed that the formation of the black bead-like structure was totally suppressed as shown in Figure 3A (detail view) and the column graph (Figure 3B). As these data have shown, there were no sclerotia produced by the Δ*Aflset1* strain, but about 110 to 290 sclerotia/cm^2^ were found from Ctrl and Δ*Aflset1*-Com strains. By qRT-PCR analysis, it was found that in absence of AflSet1 expression, the expression levels of the key sclerotia formation regulators (*nsdC*, *nsdD* and *sclR*) were significantly down-regulated (Figure 3C). All these results suggested that AflSet1 regulates the formation of sclerotia through orthodox sclerotia formation regulators.

### AflSet1 Regulates AFB1 Synthesis in *A. flavus*

**FIGURE 4:**
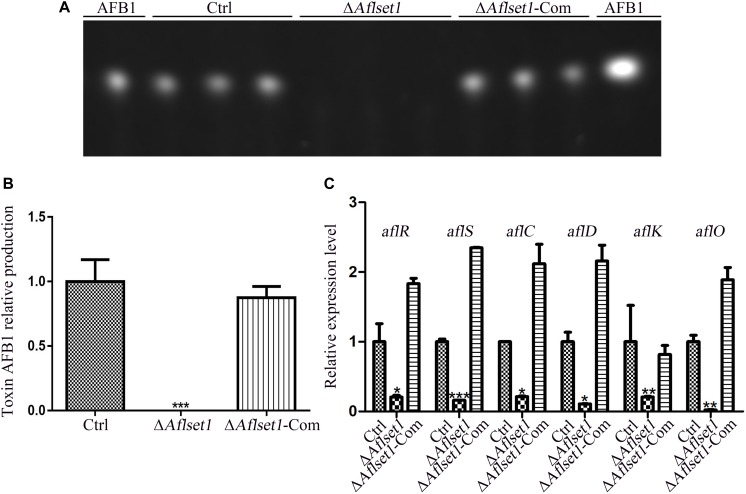
AflSet1 regulates AFB1 synthesis by orthodox aflatoxin synthesis pathway. **(A)** TLC analysis on the production of AFB1 from the fungal strains listed above. **(B)** The column graph showing the amount of AFB1 produced by these fungal strains according to the result from TLC analysis in the panel **(A)**. **(C)** The expression levels of AFB1 synthesis regulator genes (*aflR* and *aflS*), and AFB1 synthesis enzyme genes (*aflC*, *aflD*, *aflK*, and *aflO*) in the orthodox AFB1 synthesis pathway were analyzed by qRT-PCR.

*Aspergillus flavus* causes significant losses to agriculture mainly through its mycotoxin, especially AFB1. The biological function of AflSet1 in AFB1 synthesis was analyzed in this work. To do this, the fungal strains were cultured with liquid YES media at 29°C for 6 days. Aflatoxins were extracted with dichloromethane, and analyzed by thin layer chromatography with silica gel plate. As the results showed in Figures 4A,B, the AFB1 production capacity was blocked when AflSet1 was absent. To identify the pathway by which AflSet1 controls AFB1 synthesis, qRT-PCR analysis was performed, and the results showed that the expression levels of key transcriptional factor genes (*aflR* and *aflS*) and the enzyme genes (*aflC, aflD, aflK*, and *aflO*) in the orthodox aflatoxin synthesis pathway were dramatically down-regulated in Δ*Aflset1* strain compared to those of Ctrl and Δ*Aflset1*-Com strains (Figure 4C). All these results indicated that AflSet1 positively participates in the biological synthesis of AFB1 by orthodox aflatoxin synthesis pathway.

### AflSet1 Is Important for *A. flavus* Virulence in Its Colonization on Crop Kernels

**FIGURE 5:**
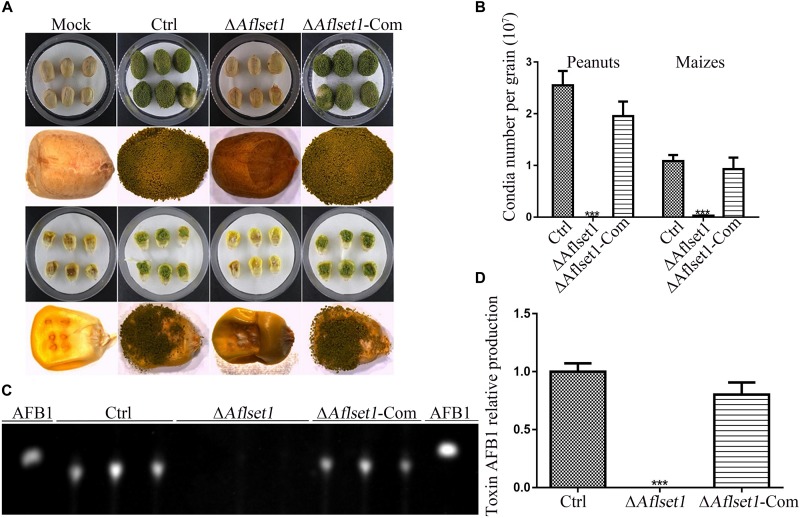
The roles of AflSet1 in the colonization of *A. flavus* on crop kernels. **(A)** Colonization of *A. flavus* strains on peanut and maize kernels. **(B)** The number of conidia on infected crop kernels in the panel **(A)** was calculated. **(C)** AFB1 production from those infected crop kernels was determined with TLC analysis. **(D)** The amount of AFB1 was calculated according to the TLC result in the panel **(C)**.

As one of the plant pathogens, *A. flavus* always contaminates various kinds of crop grains, especially peanut and maize, which wastes lots of oil and food crops and causes enormous economic losses each year (Yang et al., 2018). To evaluate the role of AflSet1 in colonization of the fungal on crop grains, we inoculated the fungal spores on the kernels of maize and peanut with the same conidia concentration and incubated at 29°C for 6 days. The results showed that the colonization ability of Δ*Aflset1* strain on both hosts (peanut and maize kernels) decreased dramatically (Figure 5A), and the Δ*Aflset1* knockout mutant fungi hardly sporulated, compared to Ctrl and Δ*Aflset1*-Com strains (Figure 5B). The AFB1 was extracted from these *A. flavus* contaminated kernels, and further detected with TLC analysis. The result showed that no detectable aflatoxins were found in Δ*Aflset1* strain, but an amount of about 0.7–1.10 (relative value) AFB1 was detected in Ctrl and Δ*Aflset1*-Com strains (Figures 5C,D). These results reflected that AflSet1 plays vital role in colonization and mycotoxin production on host kernels.

### AflSet1 Is Involved in the Regulation of Lipase Activity in *A. flavus*

Oil crops (including maize and peanut) are susceptible to *A. flavus*, and aflatoxins are always found in these oily kernels even prior to their harvest (Woloshuk and Prieto, 1998). It is interesting to explore the bio-function of AflSet1 in the activity of the fungal lipase, since AflSet1 plays a key role in the virulence of *A. flavus* during its colonization on oil kernels. In the study, fungal strains were point inoculated on PDA medium with 0.3% tributyrin (C_15_H_26_O_6_) at 29°C for 3 days. It showed that the colony diameter of Δ*Aflset1* strain on PDA medium with or without tributyrin almost kept the same, but the colony diameters of Ctrl and Δ*Aflset1*-Com strains on PDA medium were obviously bigger than those on 0.3% tributyrin PDA medium (Supplementary Figure S2A). And further calculation confirmed that the relative inhibition rate of tributyrin to the colony growth of Ctrl and Δ*Aflset1*-Com strains was significantly higher than that of Δ*Aflset1* strain (Supplementary Figure S2B). The degradation product of tributyrin catalyzed by lipase would significantly suppress the growth of microorganisms (Wielen et al., 2000). The result hinted that the absence of AflSet1 might dramatically reduce the activity of lipase in *A. flavus*.

### AflSet1 Is Involved in the Resistance of *A. flavus* to Osmotic, Oxidative and Cell Membrane Stress

**FIGURE 6:**
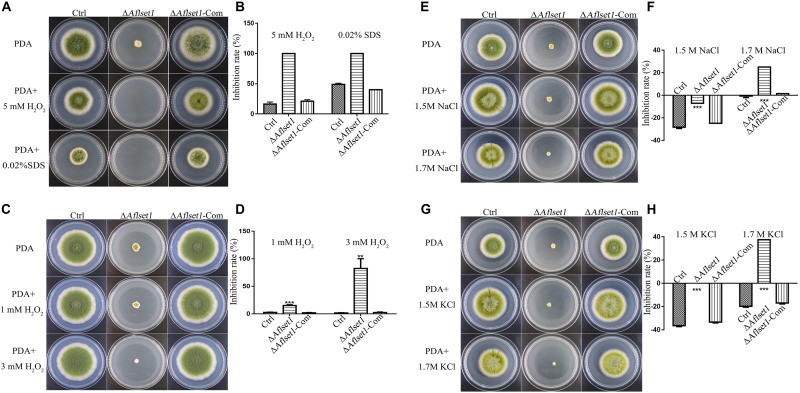
AflSet1 plays important roles in *A. flavus* against the osmotic, oxidative and cell membrane stress. **(A)**. The fungal strains (Ctrl, Δ*Aflset1* and Δ*Aflset1*-Com) were point-cultured in PDA medium with 5 mM H_2_O_2_ or 0.02% SDS. **(B)** The inhibition rate of fungal growth with 5 mM H_2_O_2_ or 0.02% SDS. **(C)** The fungal colonies were cultured on PDA medium with 1 mM H_2_O_2_ or 3 mM H_2_O_2_. **(D)** The inhibition rate of fungal growth with 1 mM H_2_O_2_ or 3 mM H_2_O_2_. **(E)** The fungal colonies were cultured on PDA medium with 1.5 M or 1.7 M NaCl. **(F)** The inhibition rate of fungal growth with 1.5 M or 1.7 M NaCl. **(G)** The fungal colonies were cultured on PDA medium with 1.5 M or 1.7 M KCl. **(H)** The inhibition rate of fungal growth with 1.5 M or 1.7 M KCl. The inhibition rate = (colony diameter without inhibitor – colony diameter with inhibitor)/colony diameter without inhibitor.

To assess the role of AflSet1 in the resistance of the fungus against surrounding stress factors, we analyzed the roles of AflSet1 in H_2_O_2_ mediated oxidative stress, SDS mediated plasma membrane stress, NaCl and KCl mediated osmotic stress, CFW mediated cell wall stress, and MMS mediated DNA stress. No evidence showed that AflSet1 is involved in the resistance of the pathogenic fungus against CFW mediated cell wall stress or MMS mediated DNA stress (Supplementary Figure S3). Under the stress of 5 mM H_2_O_2_ or 0.02% SDS, the spore germination and hyphae growth of Δ*Aflset1* were totally inhibited, and the inhibition rate of 5 mM H_2_O_2_ or 0.02% SDS to the hyphae growth of Δ*Aflset1* was 100%, which was strikingly higher than that of Ctrl (lower than 25%) or Δ*Aflset1*-Com strain (about 50%) (Figures 6A,B). When we lowered the concentration of H_2_O_2_ to 1 mM and 3 mM, the inhibition rate of H_2_O_2_ to Δ*Aflset1* was still significantly higher than that of Ctrl or Δ*Aflset1*-Com strain (Figures 6C,D). And we found that under 1.5 M and 1.7 M NaCl or KCl, the inhibition rate in Δ*Aflset1* was significantly higher than that in Ctrl and Δ*Aflset1*-Com strain (Figures 6E–H). The results showed that AflSet1 involves in the resistance of *A. flavus* against environmental stresses.

### AflSet1 Regulates Dimethylation and Trimethylation of H3K4 and Dimethylation of H3K9

**FIGURE 7:**
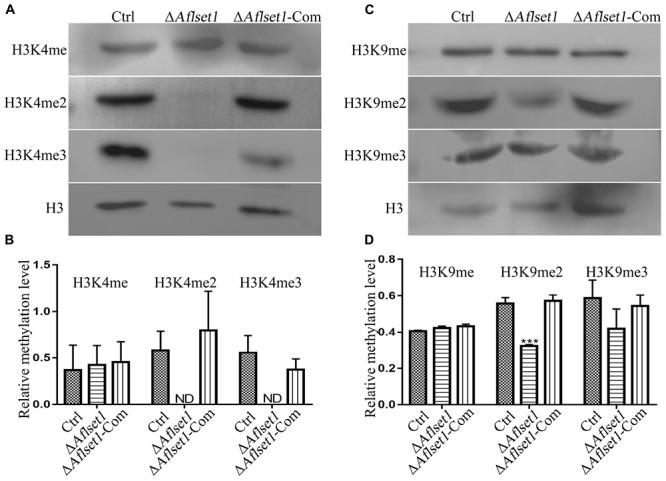
AflSet1 is involved in the H3K4me2 and -me3 and H3K9me2. **(A)** The role of AflSet1 in H3K4me2 and H3K4me3. **(B)** The column graph showing the relative methylation level of H3K4 according to the results from panel **(A)**. **(C)** The role of AflSet1 in the methylation of H3K9. **(D)** The relative methylation level of H3K9 according to panel **(C)**. The immunoblotting analysis was repeated three times.

It was reported that Set1 solely mediated H3K4 methylation in *S. cerevisiae* (Dehé and Géli, 2006). In this study, western-blotting analysis was carried out to explore the bio-function of AflSet1 in the methylation of lysine in histone H3. The proteins from fungal strains were prepared and transferred onto NC membrane after SDS-PAGE. The NC membrane was further detected with monoclonal antibodies against methylated H3K4, H3K9, and H3K36. The results showed that no band was detected on the lanes of H3K4me2 and me3, compared to Ctrl and Δ*Aflset1*-Com strains (Figure 7A). The relative methylation level of H3K4 was analyzed in Figure 7B, which showed that the absence of AflSet1 completely suppressed di- and trimethylation of H3K4, but the monomethylation of H3K4 was no obviously affected. The immunoblotting results from Figure 7C showed that H3K9me2 was obviously suppressed when AflSet1 was absent. Further statistical analysis revealed that the deletion of *Aflset1* significantly decreased the dimethylation of H3K9, but had no obvious effect on the mono- and trimethylation of H3K9 (Figure 7D). The immunoblotting analysis on H3K36 showed that AflSet1 does not participate in the methylation of this lysine position in H3 (data no shown). These results suggested that AflSet1 might regulate fungal morphogenesis and virulence via di- and trimethylation of H3K4 and dimethylaton of H3K9 in *A. flavus*.

### AflSet1 Is Accumulated in the Nucleus of *A. flavus*

**FIGURE 8:**
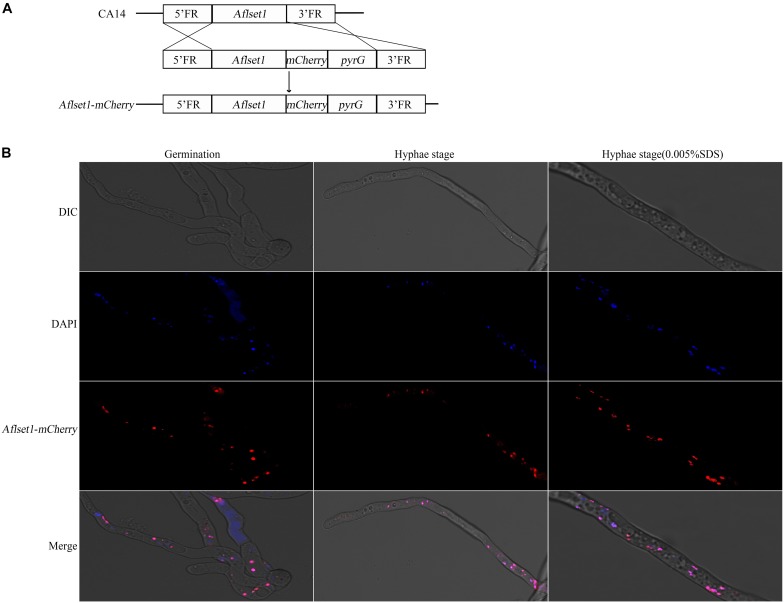
Subcellular localization of AflSet1. **(A)** The scheme of the construction strategy for *Aflset1*-*mCherry* fusion expression fungal strain. FR stands for flanking region. **(B)** Photos of AflSet1 subcellular location through fused mCherry with or without stress (0.05% SDS) at both conidia germination and hyphae stage.

As a methyltransferase, AflSet1 might be accumulated in nuclei to perform its biological function. To investigate its subcellular localization in *A. flavus*, we generated a fungal strain in which AflSet1 was tagged by mCherry at its N-terminus (Figure 8A). The AflSet1-mCherry fungal strain was cultured in YES liquid medium for 6 h (for spore germination observation) and for 12 h (for hyphae observation) at 37°C. To visualize nuclei, the mycelia samples were incubated with 1 μg/mL DAPI for 30 min. The mycelia samples were further observed under Leica SP8 confocal laser scanning microscope. The nuclei position was located by a light source with a 405 nm wavelength, and the sub-cellular location of AflSet1-mCherry was identified by a 552 nm light source. Finally, through dual-channel imaging, we confirmed that AflSet1 is accumulated in the nuclei in both spore germination and hyphae stage as shown in the left two rows in Figure 8B, and that even under the stress of SDS, AflSet1 stably stayed inside the nuclei as shown in the right two rows in Figure 8B.

### N_SET and SET Domain of AflSet1 Are Involved in Morphogenesis and AFB1 Biosynthesis in *A. flavus*

**FIGURE 9:**
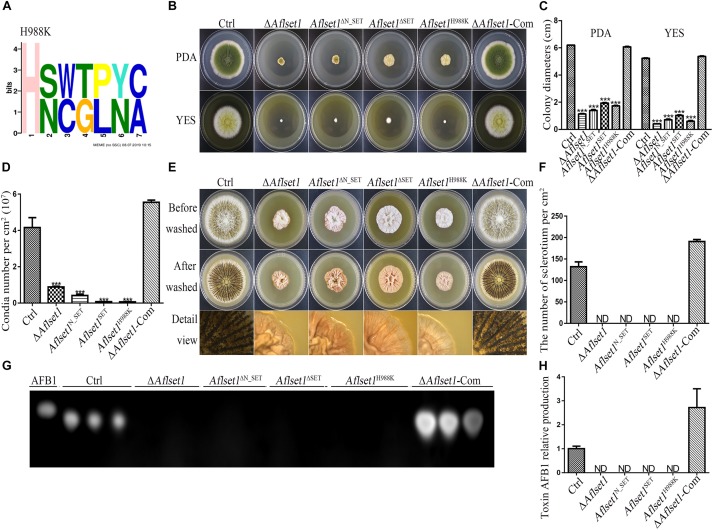
N_SET, SET domain and H988 of AflSet1 are involved in morphogenesis and AFB1 bio-synthesis in *A. flavus*. **(A)** The conserved motif with seven amino acids was identified in *A. flavus* with MEME (http://meme-suite.org.), and H988 was found to be in one of these conserved motifs. **(B)** Fungal strains (Ctrl, Δ*Aflset1*, *Aflset1*^ΔN_SET^, *Aflset1*^ΔSET^, *Aflset1*^H988K^ and Δ*Aflset1*-Com) were point-inoculated on PDA and YES agar and incubated at 37°C to observe colonial growth and conidiation. **(C)** The column graph showing the diameters of fungal colonies according to the results from panel **(B)**. **(D)** The conidia number of above fungal strains was calculated according to panel **(B)**. **(E)** Fungal strains were point inoculated on Wickerham medium and incubated for 1 week for sclerotia production. **(F)** The sclerotia number was calculated according to panel **(E)**. **(G)** The production of AFB1 from the above fungal strains was analyzed with TLC. **(H)** The column graph showing the AFB1 production from these fungal strains according to the TLC results in panel **(G)**.

By domain analysis, two important domains (N_SET and SET domain) were found in AflSet1 (Figure 1C). To illuminate the roles of N_SET and SET domain of AflSet1 in the fungal development and AFB1 synthesis, the domain deletion mutants *Aflset1*^ΔN_SET^ and *Aflset1*^ΔSET^ were constructed by knocking-out N_SET and SET domains, and further verified by sequencing (Supplementary Figure S4). Then fungal strains, including Ctrl, Δ*Aflset1*, *Aflset1*^ΔN_SET^, *Aflset1*^ΔSET^ and Δ*Aflset1*-Com, were cultured on PDA for 6 days and YES medium for 4 days. Similar to the absence of the whole AflSet1 protein, the missing of N_SET or SET domain dramatically reduced the colonial diameter and fungal conidiation (Figures 9B–D). When these fungi were grown on Wickerham medium for 1 week, it was found that the absence of N_SET or SET domain totally inhibited the sclerotia formation (Figures 9E,F). The roles of N_SET and SET domain in AFB1 bio-synthesis were also examined, and the result showed that AFB1 was not detectable by TLC analysis when either domain was removed (Figures 9G,H). All the above results indicated that N_SET and SET domains in AflSet1 play critical roles in the regulation of AflSet1 on fungal development and AFB1 production in *A. flavus*.

### H988 of AflSet1 Plays Important Roles in Colonial Growth, Sclerotia and Conidia Production and AFB1 Biosynthesis

It has been reported that H1191 in SET1 involved in the development of *F. fujikuroi* (Janevska et al., 2018). By sequence alignment, H988 in *A. flavus* was found to be corresponding with H1191 in *F. fujikuroi* (Supplementary Figure S5). To explore the biological function of H988 in AflSet1, *Aflset1*^H988K^ strain was prepared by mutation of histidine 988 to lysine according to the protocol provided in MAM (Materials and methods) and verified by sequencing (Supplementary Figure S4). Fungal strains (Ctrl, Δ*Aflset1*, *Aflset1*^H988K^ and Δ*Aflset1*-Com) were inoculated on PDA and YES agar at 37°C, and the results in Figures 9B–D showed that when histidine 988 in AflSet1 protein was substituted by lysine, fungal colonial growth and sporulation were severely suppressed, which was like what happened to Δ*Aflset1* strain. On Wickerham medium, no sclerotium was found when H988 was replaced by K, compared to Ctrl and Δ*Aflset1*-Com strains (Figures 9E,F). Further analysis with TLC showed that the replacement of H988 by K eliminated AFB1 bio-synthesis from *Aflset1*^H988K^ strain (Figures 9G,H). By MEME analysis, a conserved motif with seven amino acids was found in *A. flavus*. And H988 was found to be one of these conserved motifs, and the most conserved amino acid (Figure 9A). All these results revealed that the conserved H988 is essential in proper executing normal biological functions by AflSet1.

## Discussion

The Set1 sequence and domain architecture are conservative in fungi. As the result shown in Figure 1C, Set1 homologs in *A. oryzae*, *A. nidulans*, *F. gramincarum*, *M. oryzae*, and *S. cerevisiae* are composed of all domains found in AflSet1. Including the Set1 from *S. cerevisiae*, the Set1 proteins from fungal species are classified into a group (Figure 1B), and the SET domain is the catalytic site of Set1 (Qu et al., 2018). Set1 complex (comprised of eight subunits, including Bre2) in *S. cerevisiae* methylates H3K4, Set1 and Bre2 are required for the integrity of Set1 complex in the fungus (Roguev et al., 2001). In *A. nidulans*, the main function of Set1 is mediating the methylation of H3K4, and it is reported to initiate mitosis with kinases CDK1 and kinases NIMA during mitosis (Govindaraghavan et al., 2014). FgSet1 is highly similar to *M. oryzae* and *A. nidulans* Set1 (Figure 1B), and it is essential for mono-, di-, and trimethylation of H3K4 in *F. graminearum* (Liu et al., 2015). In *A. flavus*, AflSet1 (XP_002375305.1) is composed of 1024 amino acid residues coding by two exons in a 3609 bp DNA fragment. In the present study, bioinformatics analysis showed that all Set1 homologs from fungi contains: a RRM_SF, a SET_assoc, a N_SET, a SET and a PostSET domain (Figure 1C), and the minimum identity is 63.99% (99% Query Cover) between AflSet1 and Set1 from *A. nidulans* in *Aspergillus* spp., which suggests that Set1 is conservative, and might implement important biological function in this filament fungi.

Set1 is involved in fungal growth. The substitute of arginine for lysine 4 in H3 (H3K4R) hindered the methylation of H3 substrate by Set1 complex, which caused significant growth defects to *A. nidulans* mutants whose function of NIMA mitotic kinase were impaired (Govindaraghavan et al., 2014). It was also found that the deletion of *set1* gene or histone H3 mutations at lysine four delayed the growth of *S. cerevisiae* (Briggs et al., 2001). Our study revealed that the absence of AflSet1 dramatically inhibit the growth of *A. flavus*, which suggests that AflSet might be involved in the growth of fungal hyphae through NIMA mitotic kinase (Figures 2A,B). In *F. graminearum*, the absence of FgSet1 significantly reduced hyphae growth and virulence of the fungus, but the sporulation of the fungus was not obviously affected (Liu et al., 2015). In this study, we found that AflSet1 involves in sporulation regulation in *A. flavus* (Figures 2A,C), which is different from FgSet1. Transcriptional factor *brlA* promotes the switch from vegetative apical growth to conidiophore budding growth, and *abaA* is responsible for the development and function maintenance of phialides (Adams et al., 1988; Sewall et al., 1990). By q-PCR analysis, it was found that AflSet1 up-regulates fungal sporulation through the pathway of *brlA* and *abaA* (Figure 2D). AflSet1 also up-regulates sclerotia formation (Figures 3A,B). GATA-type zinc finger transcriptional factor NsdD and C_2_H_2_ zinc finger DNA binding protein NsdC regulate the formation of the sexual reproduction structure – cleistothecia in *A. nidulans*, and are involved in the development of sclerotia (ascospores are found in the structure) in *A. flavus* and *A. parasiticus* (Cary et al., 2012; Horn et al., 2014). SclR (sclerotium regulator), a basic-region helix-loop-helix transcriptional factor, was reported to promote the production of sclerotia (Jin et al., 2011). Through q-PCR analysis, the study revealed that AflSet1 controls the formation of sclerotia in *A. flavus* via positive regulation of transcriptional factor NsdC, NsdD, and SclR (Figure 3C).

AflSet1 is also one of the critical factors in AFB1 biological synthesis. The deletion of *Fgset1* from *F. graminearum* blocks the production of DON (Liu et al., 2015). Similar result was found in *Aflset1* deletion Δ*Aflset1*, and no detectable AFB1 was produced after 6 days culture in liquid YES and even on crop kernels by Δ*Aflset1*, compared to Ctrl and Δ*Aflset1*-Com strains (Figures 4A,B), which revealed that AflSet1 is involved in secondary metabolism of *A. flavus*. In orthodox aflatoxin biosynthesis gene cluster, the pathway specific transcriptional factor – AflR is indispensable in activation of most structure genes in the gene cluster by binding to 5′-TCGN5CGR-3′ sequence in promoter regions of these structure genes. Coded adjacent to *aflR* gene in aflatoxin gene cluster, the regulator AflS (also called AflJ) is transcribed divergently from *aflR* gene (Chang, 2003). The *aflS* gene mutants down-regulate 5- to 20-fold some aflatoxin structure genes (including *aflC*, *aflD*, *aflM* and *aflP*), and lose the ability to synthesize aflatoxin intermediates (Chang, 2003; Yu, 2012). The structure genes (*aflC, aflD, aflK*, and *aflO*) are responsible for the conversion of acetate to NOR, NOR to AVN, VHOH to VER B, and DMST to ST, respectively (Chang, 2003; Yu, 2012). Our work showed that AflSet1 participates in the regulation of mycotoxin production, and regulates the biological synthesis of AFB1 by AflR and AflS mediated orthodox aflatoxin pathway (Figure 4C).

It has been reported that FgSet1 is necessary for full virulence of fungus (Liu et al., 2015). This study revealed that few hyphae were produced on the surface of kernels by Δ*Aflset1* strain, and both the conidiation and AFB1 biological synthesis were blocked when *Aflset1* was deleted (Figure 5). Fungal colonization capacity is bound up with the activity of hydrolytic enzyme, in which lipase is important for fungal development and secondary metabolism (Gallo et al., 2010; Toth et al., 2017). One tributyrin molecule could be rapidly hydrolyzed by lipases to produce three molecules of butyrate, which means that the lipase degrades tributyrin and releases lots of butyrate, and high concentration of butyrate suppresses the growth of microorganisms (Wielen et al., 2000; Cresci et al., 2013). By analyzing lipase activity with tributyrin in the study, it is suggested that the absence of AflSet1 might dramatically decrease the lipase activity in Δ*Aflset1* strain as shown in Supplementary Figures S2A,B, in which the growth rate of Δ*Aflset1* strain colony was not affected, compared to that of Ctrl and Δ*Aflset1*-Com strains. The supposed roles of AflSet1 in digestive enzymes are worthy for further exploration, which may explain why few hyphae and conidia were produced by Δ*Aflset1* strain on crop kernels as shown in Figure 5A.

As a mark of transcriptional activation, the H3K4 methylation is conserved from yeast to human, and it is thought that H3K4 methylation results from the activity of methyltransferase Set1/COMPASS (Shilatifard, 2012; Soares et al., 2017; Jeon et al., 2018). In *F. graminearum*, FgSet1 is the methyltransferase responsible for the mono-, di- and trimethylation of H3K4 (Liu et al., 2015). To examine the biological function of AflSet1 in the methylation of histone in *A. flavus*, western-blotting analysis was performed in the study. The results of the immunoblotting analysis showed that different from FgSet1, AflSet1 dominantly regulates the di- and trimethylation of H3K4, and partially takes part in the dimethylation of H3K9, but no monomethylation of H3K4 or mono- and trimethylation of H3K9 (Figure 7). The results reflected that the methylation functions of Set1 are just relatively (not absolutely) stable among different fungal species. Di- and tri-methylation at H3K4 are typical epigenetic signature for gene activation (Jambhekar et al., 2019). The previous study showed that the methylation of H3K9 is associated with transcriptional repressed loci, and that different from H3K9me3 which is associated with transcriptional silence, H3K9me2 is associated with transcriptional activation (Liu et al., 2015; Jih et al., 2017). H3K9me2 loci contain euchromatic transcription associated modifications, and couple transcript degradation via RNAi to the establishment of H3K9 methylation loci (Jih et al., 2017). The results of the study supposed that, mainly by catalyzing the di- and trimethylation of H3K4 and involving the dimethylation of H3K9, AflSet1 up-regulated fungal development, secondary metabolism, and virulence principally via positive regulation of orthodox conidiation, sclerotia formation, and aflatoxin biosynthesis pathways. As one of the important epigenetic factors, Set1 is reported to catalyze the methylation of H3K4 in nucleus (Roguev et al., 2001; Liu et al., 2015). To further confirm the epigenetic biological function of the histone lysine methyltransferase – AflSet1, sub-cellular location analysis with mCherry was carried out in this study. The results showed that AflSet1 is stably accumulated in nucleus in all life stages (during spore germination and hyphae growing stages), even under the stress of SDS (Figure 8B). The immunoblotting and sub-cellular location analysis indicated that AflSet1 is an important epigenetic factor that stably accumulates in nucleus to regulate the morphogenesis, AFB1 biological synthesis and virulence of *A. flavus*.

The catalytic SET domain is located at the C-terminal of SET1, and it is reported that the N_SET domain of Set1 is required for the stability of Set1 in yeast (Thornton et al., 2014; Qu et al., 2018). N_SET domain in Set1 is essential for H3K4, and the deletion of N_SET domain would diminish the global methylation of H3K4 in yeast (Kim et al., 2013). To explore the roles of above both domains in the biological function of AflSet1, SET domain mutant (*Aflset1*^ΔSET^) and N_SET domain mutant (*Aflset1*^ΔN_SET^) strains were constructed in this study. The results showed that like what happened to *Aflset1*^ΔSET^, the absence of N_SET domain sharply reduced the rate of hyphae growth, sporulation, sclerotia production and AFB1 biosynthesis in *A. flavus*, which reflected that both domains are the most critical domains to fulfill the proper biological function of AflSet1. In *Fusarium fujikuroi*, it is reported that point mutation of H1191 to K in SET1 inhibited the methylation of H3K4, and decreased the growth of the pathogenic fungus, which resembles the situation found in *F. fujikuroi* SET1 deletion strain (Janevska et al., 2018). By sequence alignment, H988 in *A. flavus* was found corresponding to the H1191 in *F. fujikuroi*. *Aflset1*^H988K^ was generated and further analysis showed that H988 plays critical roles in mycelia growth, conidiation, sclerotia formation and AFB1 biosynthesis, which is similar to what is found in Δ*Aflset1* strain (Figure 9). All these results suggested that both SET and N_SET domains are almost indispensable in proper implementation of the biological function of AflSet1, and H988 is one of the most critical amino acids in the enzyme active site in AflSet1 in *A. flavus*.

**FIGURE 10:**
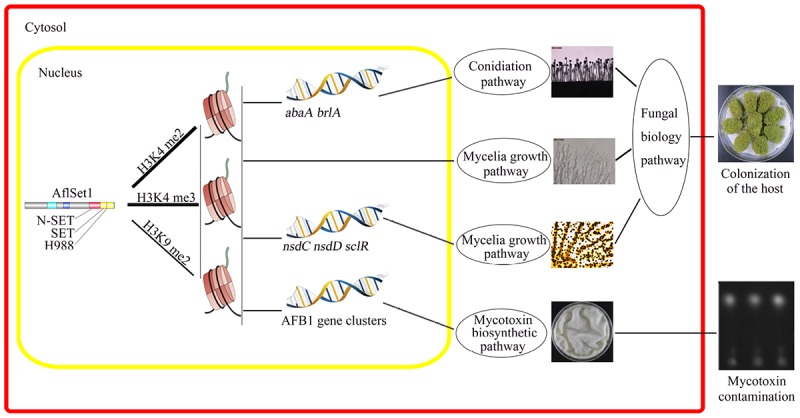
The model showing the pathways by which AflSet1 regulates the morphogenesis, AFB1 production and virulence of *A. flavus*. AflSet1 mediates the di- and trimethylation at H3K4 and is involved in the dimethylation at H3K9 in fungal nuclei, mainly via which AflSet1 regulates fungal conidiation through transcriptional factors BrlA and AbaA, controls sclerotia formation by NsdC, NsdD and SclR, promotes AFB1 synthesis by orthodox aflatoxin pathway, and plays an important role in the colonization of the pathogen to crop kernels by up-regulating hyphae growth, sporulation and sclerotia formation.

In conclusion, our study reveals that AflSet1 plays critical roles in fungal morphology, secondary metabolism and virulence mainly through its methyltransferase activity on all H3K4me2 and -me3 and part of H3K9me2 (Figure 10). Importantly, our study also provides a basis to illuminate the biological function of histone methyltransferase in pathogenic fungi, and paves a new way for the control of the contamination of pathogenic fungi to crops.

## Data Availability Statement

The raw data supporting the conclusions of this article will be made available by the authors, without undue reservation, to any qualified researcher.

## Author Contributions

ZZ designed and wrote the manuscript, took part in all experiments of the whole project, and provided funds support. ShW provided the help in projection designation, manuscript correction, and part of fund support. YL took part in all experiments, and constructed Aspergillus flavus deletion strain in the study, took part in the experiments on stress resistance of *A. flavus*. MZ took part in the experiments on aflatoxin production analysis, and morphogenesis analysis. RX took part in the experiments of peanut seeds and corn grains model construction. FZ took part in the experiments of corn grains model construction. SeW provided the help in the analysis of the data. XP provided the help to construct the domain deletion and point mutants.

## Conflict of Interest

The authors declare that the research was conducted in the absence of any commercial or financial relationships that could be construed as a potential conflict of interest.

## Footnotes

1 https://www.ncbi.nlm.nih.gov/

2 http://dog.biocuckoo.org
