# Supplementary material for: The Methyltransferase AflSet1 Is Involved in Fungal Morphogenesis, AFB1 Biosynthesis, and Virulence of Aspergillus flavus
Source: Front Microbiol. 2020 Feb 18;11:234. doi: 10.3389/fmicb.2020.00234 (PMC7040179; doi:10.3389/fmicb.2020.00234)
Supplement: Supplementary file 1 [file Data_Sheet_1.pdf]

Table S1. The primers used in this study

Primer	DNA Sequence (5'→ 3')	Fragment amplified
Aflset1-p1	CACCTCTCAGCATCCAATCCTCTAGCATTG	For 5'FR amplification in Δ*Aflset1* strain preparation
Aflset1-p2	GGGTGAAGAGCATTGTTTGAGGCAGACCAGCTGCG ATCAGAAGGC	
Aflset1-p3	GCATCAGTGCCTCCTCTCAGACCTCTGCAGATGAG GAGCTGACGTATGACTAC	For 3'FR in Δ*Aflset1* strain preparation
Aflset1-p4	ATTACAAGCTTGGGTTCCCCAAAATTCTGGA	
Aflset1-p5	GCCTCAAACAATGCTCTTCACC	For *pyrG* amplification in Δ*Aflset1* strain preparation
Aflset1-p6	GTCTGAGAGGAGGCACTGATGC	
Aflset1-p7	GTGGATAGTAGTTTGCGGTGTATACAGCCAAG	Nesting primers for fragments fusion in Δ*Aflset1* preparation
Aflset1-p8	GCATGAGTACTTGATCAAAGAGGGGATCG	
Aflset1-p9	CTTTGCAGACTTCTTCCCTACTGCGCCGTC	To amplify a fragment inside *Aflset1* ORF
Aflset1-p10	TTATGCGCTTTTGCCAAACTCACCTCTCTCG	
Aflset1-q-F	GAGCCCATTGTCGATGACGAC	To amplify a fragment from *Aflset1* ORF in qRT-PCR
Aflset1-q-R	AGCCGACAAATTCCCAAC	
P1020	ATCGGCAATACCGTCCAGAAGC	*pyrG* testing primers used in Δ*Aflset1* strain verification
P801	CAGGAGTTCTCGGGTTGTCG	
Probe-F	CACCTCTCAGCATCCAATCCTCTAGCATTG	To amplify probe in Southern-blotting analysis
Probe-R	GGGTGAAGAGCATTGTTTGAGGCAGACCAGCTGCG ATCAGAAGGC	
Aflset1-C-p1	CCAATTACTATTACGGGCCTCTTACCTCG	To amplify 5'FR in Δ*Aflset1*-Com strain preparation
Aflset1-C-p2	GGGTGAAGAGCATTGTTTGAGGCCCTCAAGCAGTT TAGGCAGTCAGCTC	
Aflset1-C-p3	GCATCAGTGCCTCCTCTCAGACCTGTTGTGCAAAG ACTCAGAGGAAGGTAG	To amplify 3'FR in Δ*Aflset1*-Com strain preparation
Aflset1-C-p4	CCACATCACGATTTCCACATGCTCTCAGG	

Aflset1-RFP-P1	GTATTCGAAAGGCTCGTCATCT	For 5'FR amplification in *Aflset*1-RFP strain preparation
Aflset1-RFP-P2	CCTCGCCCTTGCTCACCATTGCGCTTTTGCCAAACTCACCT	
RFP-F	ATGGTGAGCAAGGGCGAGG	To amplify RFP in *Aflset*1-RFP strain preparation
RFP-R	CTACTTGTACAGCTCGTCCAT	
Aflset1-RFP-P3	GCATCAGTGCCTCCTCTCAGACCTCTGCAGATGAGGAGCTGACGTATGAC	To amplify 3'FR in *Aflset1*-RFP strain preparation
Aflset1-RFP-P4	AGCAAATACACACATCGTGAATGGCCAGC	
Aflset1-pN1	GTGAAGTCACGCATTGCTGCACCAG	For 5'FR amplification in *Aflset1*$^{N\_SET}$ strain preparation
Aflset1-pN1-R	GATTATTGCGTCGTCGTCATCGACAATGG	
Aflset1-pN2	CCATTGTCGATGACGACGACGCAATAATC CGCTTCAATCAGCTGAAGAAACGAAAGAAGC	Primes used in N_SET deletion in *Aflset1*$^{N\_SET}$ strain preparation
Aflset1-pN2-R	GGGTGAAGAGCATTGTTTGAGGCTTATGCGCTTTTGCCAAACTCACCTCTCT	
Aflset1-pN4-F	GCATCAGTGCCTCCTCTCAGACCTCTGCAGATGAGGAGCTGACGTATGAC	To amplify 3'FR in *Aflset1*$^{N\_SET}$ strain preparation
Aflset1-p4	AGCAAATACACACATCGTGAATGGCCAGC	
Aflset1-pN8-F	GAATTGATGATTCGTTCGGGACACCGGAC	Nesting primers used in *Aflset1*$^{N\_SET}$ preparation
Aflset1-p8	CCTCTACTGTCTCTGCCCTTACCTATTCCACC	
Aflset1-pN8-F	GAATTGATGATTCGTTCGGGACACCGGAC	To amplify 5'FR in *Aflset1*^{SET} strain preparation
Aflset1-pS1-R	TTATGCGCTTTTGCCAAACTTTCGTTTCTTCAGCTGATTGAAGCGAAGGA	
Aflset1-*pyrG*-F	TTTGGCAAAAGCGCATAAGCCTCAAACAATGCTCTTCACCC	For *pyrG* amplification used in *Aflset1*^{SET} strain preparation
pyrG-R	GTCTGAGAGGAGGCACTGATGC	
Aflset1-PS-4-F	GCATCAGTGCCTCCTCTCAGACCTCTGCAGATGAGGAGCTGACGTATGAC	To amplify 3'FR in *Aflset1*^{SET} strain preparation
Aflset1-p4	AGCAAATACACACATCGTGAATGGCCAGC	
Aflset1-PS-8-F	GGAGTCTGATGATGGCGACGGCTTTG	Nesting primers used in *Aflset1*^{SET} preparation
Aflset1-p8	CCTCTACTGTCTCTGCCCTTACCTATTCCACC	

Aflset1-H988K-1-F	ATCCTTCCAAAAGAGAGTAATGATAACC	To amplify 5'FR in *Aflset1*^{H988K} preparation
Aflset1-H988K-1-R	GGCAGTGCAATTCGGTGTACAGCTtttGTTGATAAACCTAGCAATGCCC	
Aflset1-H988K-2-F	GGGCATTGCTAGGTTTATCAACAAAAGCTGTACACCGAATTGCACTGCC	Primers designed to change H988 to K988
Aflset1-H988K-2-R	GGGTGAAGAGCATTGTTTGAGGCGCAGACAGAGCGAGAAGTCCCGTGCACAGA	
Aflset1-H988K-3-F	GCATCAGTGCCTCCTCTCAGACCTGCCTCTGTTGCCCCCTCTTTATGT	To amplify 3'FR in *Aflset1*^{H988K} strain preparation
Aflset1-H988K-3-R	GTCCCTCGGATTGATTGG	
N-set-F	ATGGTATTGATGTTACGTCCTC	Used with P801 in *Aflset1*$^{\Delta N\_SET}$ strain verification
set-F	GGAAATCAAAGCCCTCAA	Used in *Aflset1*$^{\Delta SET}$ strain verification with P801
H988K-F	AGGAAAGCATTGGCCGCTTGGGCA	Used in *Aflset1*^{H988K} strain verification
H988K-R	GTGGGACCCTTTCGCGGAGGACTGG	

FR：Flanking region.

Table S2. The primers used for q-PCR analysis

Primer	DNA Sequence (5’→ 3’)	Target gene
brlA-F	GCCTCCAGCGTCAACCTTC	*brlA*
brlA-R	TCTCTTCAAATGCTCTTGCCTC	
abaA-F	TCTTCGGTTGATGGATGATTTC	*abaA*
abaA-R	CCGTTGGGAGGCTGGGT	
nsdC-F	GCCAGACTTGCCAATCAC	*nsdC*
nsdC-R	CATCCACCTTGCCCTTTA	
nsdD-F	GGACTTGCGGGTCGTGCTA	*nsdD*
nsdD-R	AGAACGCTGGGTCTGGTGC	
sclR-F	CAATGAGCCTATGGGAGTGG	*sclR*
sclR-R	ATCTTCGCCCGAGTGGTT	
aflC-F	GTGGTGGTTGCCAATGCG	*aflC*
aflC-R	CTGAAACAGTAGGACGGGAGC	
aflP-F	CGATGTCTATCTTCTCCGATCTATTC	*aflP*
aflP-R	TCTCAGTCTCCAGTCTATTATCTACC	
aflO-F	CTTTCGGCAGTGACCTAACC	*aflO*
aflO-R	TCTTGAACTATAAGGCGACCA	
aflR-F	AAAGCACCCTGTCTTCCCTAAC	*aflR*
aflR-R	GAAGAGGTGGGTCAGTGTTTGTAG	
aflS-F	GCTCAGACTGACCGCCGCTC	*aflS*
aflS-R	GCTCAGACTGACCGCCGCTC	
actin-F	ACGGTGTCGTCACAAACTGG	*actin*
actin-R	CGGTTGGACTTAGGGTTGATAG	

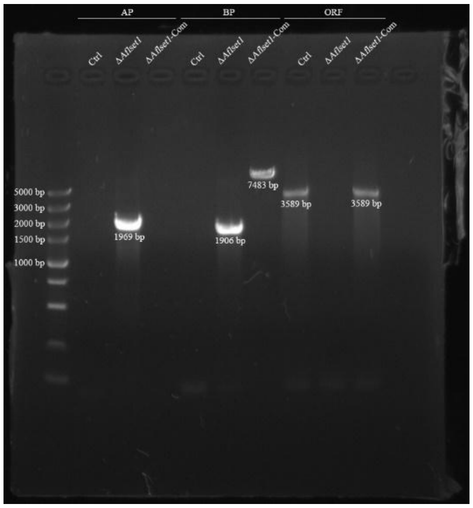

Figure S1. The verification of Δ*Aflset1* andΔ*Aflset1*-Com strains with diagnostic PCR.

The constructed Δ*Aflset1* andΔ*Aflset1*-Com strains were validated by PCR with genomic DNA as template. DNA fragment AP was amplified with primer p1 and p801, BP with primer p1020 and p4, and ORF with primer p9 and p10 as shown in **Figure 1A**.

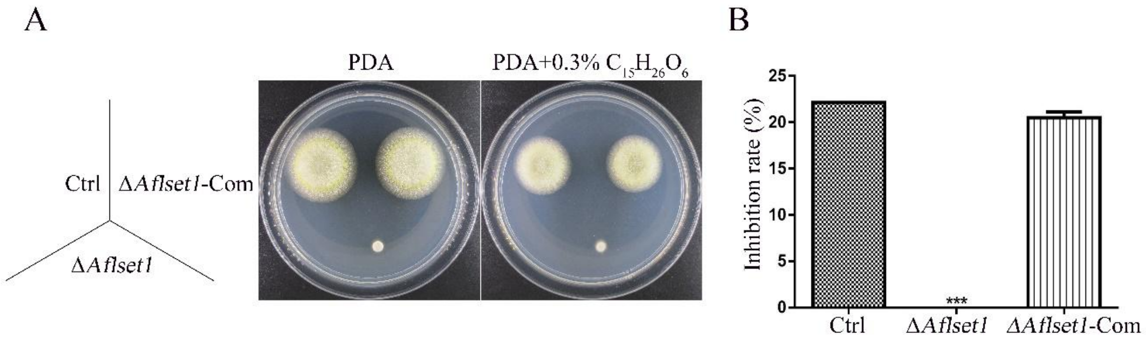

Figure S2. AflSet1 takes part in the regulation of lipase activity.

A. Fungal strains were point cultured on PDA medium with 0.3% tributyrin ($C_{15}H_{26}O_6$). **B**. The column graph showing the relative inhibition rate of tributyrin to fungal strains according to the result of panel "A". The relative inhibition rate = (Colony diameter on PDA medium - Colony diameter on PDA with 0.3% tributyrin)/ Colony diameter on PDA.

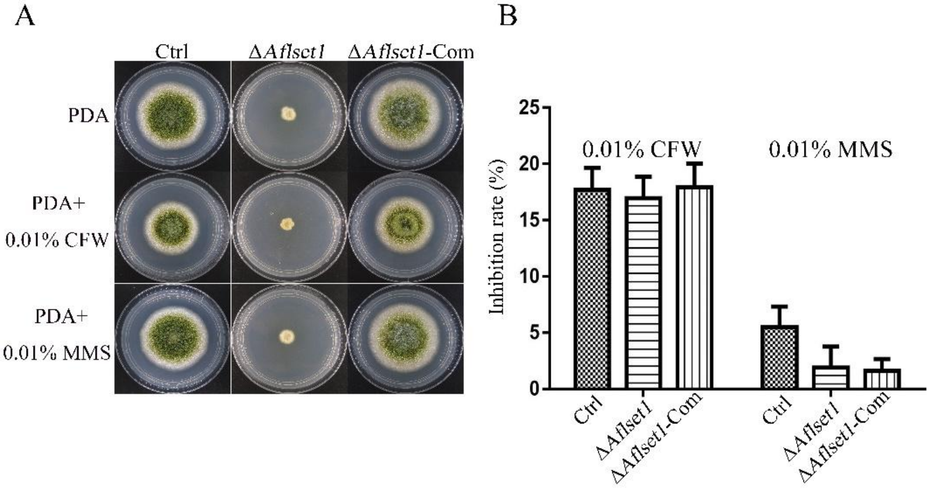

Figure S3. The roles of AflSet1 in CFW and MMS mediated stresses.

A. The pathogenic fungi (Ctrl, Δ*Aflset1* and Δ*Aflset1*-Com) were point-cultured on PDA medium with 0.01% CFW or 0.01% MMS. **B.** The inhibition rate of fungal growth under 0.01% CFW or 0.01% MMS.

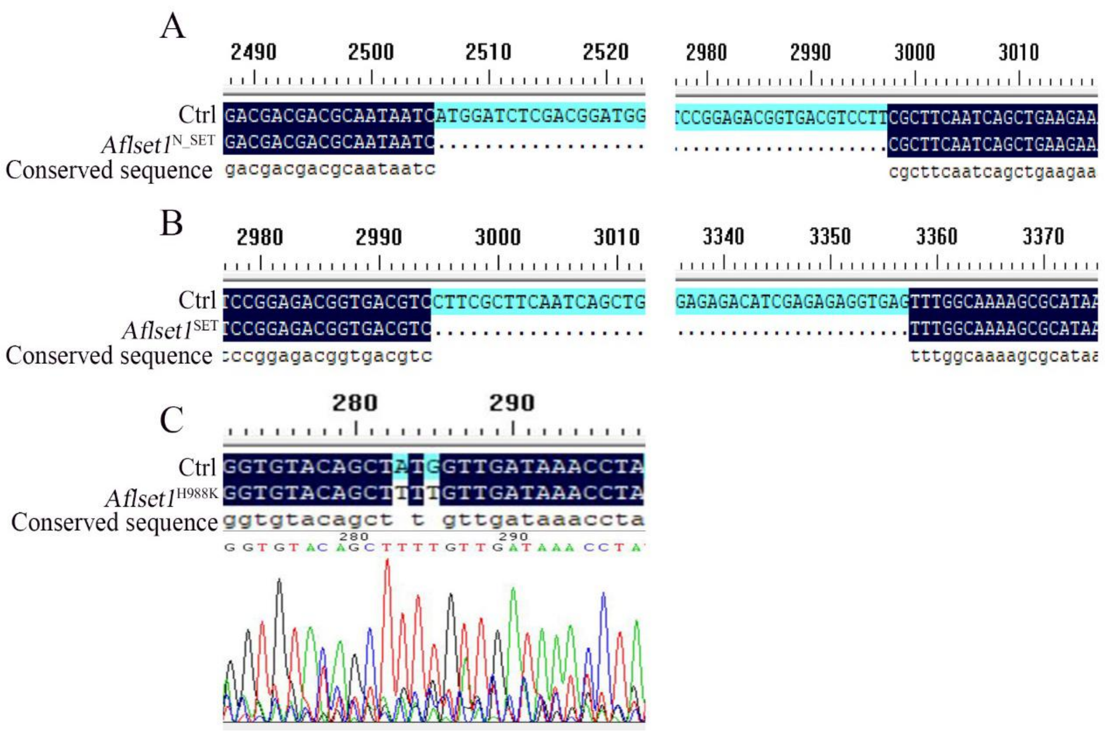

Figure S4. The sequencing results of *Aflset1*$^{\Delta N\_SET}$, *Aflset1*$^{\Delta SET}$ and *Aflset1*^{H988K} fungal strains.

A. The DNA sequence was amplified by primer N-set-F and P801 (**Table S1**) with genomic DNA of *Aflset1*$^{\Delta N\_SET}$ as template, and was sequenced by BioSune (Shanghai, China), and aligned with the sequence from the GenBank in ncbi (https://www.ncbi.nlm.nih.gov/) through DNAMAN software. **B.** With genomic DNA of *Aflset1*$^{\Delta SET}$ as template, the DNA sequence was amplified by primer set-F and P801 (**Table S1**), and was sequenced, and the amplified sequence was further aligned with the sequence from the GenBank. **C.** With genomic DNA of *Aflset1*^{H988K} as template, the DNA sequence was amplified by primer H988K-F and H988K-R (**Table S1**), and the amplified DNA sequence was sequenced and aligned with the sequence from the GenBank.

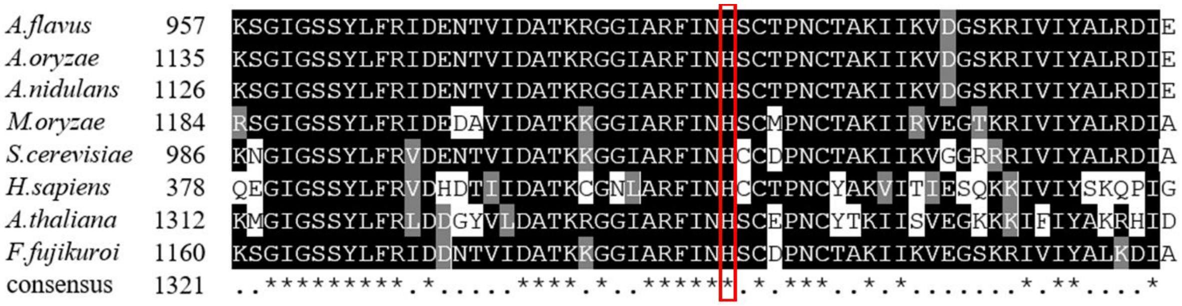

Figure S5. Amino acid alignment of AflSet1. The alignmemt of AflSet1 with other 7 homologs from *A. oryzae*, *A. nidulans*, *M. oryzae*, *S. cerevisiae*, *H. sapiens*, *A. thaliana* and *F. fujikuroi*. By this analysis, H1191 in SET1 of *F. fujikuroi* was identified to be the H988 in AflSet1 of *A. flavus*. Clustal X was used in this analysis**.**
